# Influence of Solder Mask on Electrochemical Migration on Printed Circuit Boards

**DOI:** 10.3390/ma17174242

**Published:** 2024-08-27

**Authors:** Markéta Klimtová, Petr Veselý, Iva Králová, Karel Dušek

**Affiliations:** Department of Electrotechnology, Faculty of Electrical Engineering, Czech Technical University in Prague, Technická 2, 160 00 Prague, Czech Republic; veselp13@fel.cvut.cz (P.V.); kraloiva@fel.cvut.cz (I.K.); dusekk1@fel.cvut.cz (K.D.)

**Keywords:** electrochemical migration, solder mask, dendrites, water drop test, thermal humidity bias test

## Abstract

Electrochemical migration (ECM) on the surface of printed circuit boards (PCBs) continues to pose a significant reliability risk in electronics. Nevertheless, the existing literature lacks studies that address the solder mask and solder pad design aspects in the context of ECM. Therefore, the objective of this study was to assess the impact of solder mask type with varying roughness and solder pad design on the susceptibility to ECM using a water drop test and thermal humidity bias test. Hot air solder leveling-coated PCBs were tested. Furthermore, the ECM tests were conducted on PCBs with applied no-clean solder paste to evaluate the influence of flux residues on the resulting ECM behavior. The results indicated that the higher roughness of the solder mask significantly contributes to ECM inhibition through the creation of a mechanical barrier for the dendrites. Furthermore, lower ECM susceptibility was also observed for copper-defined pads, where a similar effect is presumed. However, the influence of the no-clean flux residues can prevail over the effects of the solder mask. Therefore, the use of a rough solder mask and a copper-defined pad design is recommended if the PCB is to be washed from flux residues after the soldering process.

## 1. Introduction

Electronics manufacturing technology is constantly evolving and moving forward. One of the key trends in electronics is miniaturization. A higher density of assembled components, which is accompanied by a reduction in the distance between the conductive traces or terminals of components, increases the risk of issues related to electrochemical migration (ECM) [1,2]. Exposure to harsh (humid, polluted) environments also increases the chance of ECM, especially when the printed circuit board (PCB) is in direct contact with the environment [3].

ECM is a corrosion phenomenon that occurs due to the presence of an environment with humidity above a critical level and applied bias voltage, leading to a decrease in surface insulation resistance between adjacent electrodes or even their short circuit [4,5]. ECM can decrease the life span of electronic devices and can lead to their temporary or even permanent failure [6].

Predisposition of the ECM is the presence of an electric field and a continuous layer of water (or another liquid solution allowing movement of ions) between two adjacent electrodes [7]. The whole ECM process consists of four stages. The first stage is water condensation and the formation of an electrolyte layer. When the electrolyte covers both electrodes, the electric field causes the anode to start dissolving, producing positive metal ions that then migrate toward the cathode. In the last stage, metal ions are deposited on the cathode, creating conductive filaments called dendrites that grow back to the anode. When the dendrite reaches the anode, a short circuit occurs [8,9].

ECM is influenced by many factors, and their understanding is essential for the development of strategies to mitigate ECM and increase the longevity of electronic systems. Among these factors are ambient conditions (temperature, relative humidity) [10,11], electrode material [12,13,14,15], electric field [16,17,18], distance of the electrodes [19], thickness of the electrolyte layer [20], or contamination, such as flux residues [21,22,23,24]. Due to the hygroscopic nature of the flux substances (e.g., weak organic acid, halogenides), condensation is promoted, and the conductivity of the electrolyte layer may also be increased, leading to a more frequent occurrence of ECM [17,25,26]. The flux residues have the potential to attract surrounding contamination, such as dust [27]. On the other hand, Ambat et al. [23] showed that depending on the nature of the contained acid, flux residues can either act as an ECM activator or an inhibitor. Some flux residues can suppress anodic dissolution and prevent ECM occurrence.

Electrode material also plays a significant role in the ECM. Each element or solder alloy exhibits distinct ECM behavior and susceptibility. Not all metals are capable of migrating; the metals that have received the most attention are tin, copper, silver, nickel, and lead, among others. Although the first documented and studied migration was of silver, due to its high susceptibility to migration, nowadays, ECM is often studied on tin solder alloys, which comprise nearly 95% tin [28,29,30,31,32]. Yu et al. [33] focused on comparing different solder alloys consisting of tin. It was found that when the lead-free solder alloy was subjected to ECM regardless of whether it was Sn-Ag or Sn-Ag-Cu, the migrating metal was consistently tin. It was also discovered that if Ag or Cu are added to tin solder alloy, they create intermetallic compounds (IMC) with tin, which serves to suppress ECM [11,19].

Additionally, the susceptibility to ECM is also affected by the surface area between the electrodes, which can be covered with a solder mask. The purpose of a solder mask on PCB is to prevent solder wetting and, thereby, the formation of solder bridges, further prevent oxidation, reduce contamination, inhibit whisker growth, etc. [34,35]. According to He et al. [25] and Bušek et al. [10], solder masks should also suppress the ECM. Both works examined the influence of the presence of a solder mask on ECM, with its presence shown to reduce the risk of ECM in both cases. He et al. [25] performed a thermal humidity bias test (THBT) with Sn-37Pb and Sn-3.0Ag-0.5Cu solders. The test duration was more than 1000 h, and the test parameters were 40 V, 65 °C, and 88% RH. During the test, surface insulation resistance (SIR) on the PCBs without a solder mask dropped, compared to the PCBs with a solder mask, where the SIR was higher and remained stable. The appearance of dendrites was more frequent on the PCBs without a solder mask; the failure occurred in every case within 110 h. On the contrary, the presence of dendrites was rare on the PCBs with a solder mask. Bušek et al. [10] performed a direct deposition of the electrolyte on the test pattern and examined Sn-3.0Ag-0.5Cu solder on various surface materials (bare copper, ENIG, HAL, and OSP). During the test, which lasted 10 min, the bias voltage of 32 V was applied, and the dendrite growth was optically inspected. Their results confirmed that the solder mask effectively suppresses ECM.

Concerning surface roughness, it has been proven that higher surface roughness (pores, pits, and scratches) leads to faster electrolyte formation due to more localized regions for water adsorption [25]. Piotrowska et al. [36] claimed that the solder mask parameters, such as chemical composition and surface morphology, should be considered regarding corrosion. They have examined five different solder masks. It has been proven that condensation of water is faster on a rough solder mask than on a smooth solder mask. Additionally, the study identified the type of filler utilized in solder masks and the prevalence of bulk defects as factors that also influence the condensation process. Medgyes et al. [37] studied ECM on different substrates (FR4 and polyimide (PI)) and on immersion silver surface finish. These two different substrate materials were subjected to the thermal humidity bias test and water drop test (WDT). The THBT results showed a higher condensation intensity on FR4 due to its porous and rough surface and higher thermal diffusivity. However, the WDT found no differences in substrate materials. On the other hand, Noh et al. [18] examined the same surface materials (FR4, PI), where the difference in the substrates was found while performing a WDT. FR4 demonstrated higher susceptibility to ECM than PI due to its higher moisture absorption and lower surface insulation.

Previous research on the relationship between the solder mask and ECM has primarily focused on a single type of solder mask, with an emphasis on comparing samples with and without a solder mask. Piotrowska et al. [36] studied the various types of masks only in terms of water condensation. Noh et al. [18] and Medgyes et al. [37] only studied the influence of different substrate materials. In our previous research [38], we investigated the impact of improper solder mask application on electrochemical migration. Our findings indicated that the improper application of solder masks may result in reliability issues caused by residual contamination on PCBs. The findings of this study reinforce the necessity for further investigation into the relationship between the various types of properly applied solder masks and ECM, which was one of this work’s primary objectives. In contrast to our previous study, this study focused on the evaluation of the specific solder mask properties with regard to their chemical composition, surface roughness, and surface wettability.

Furthermore, no previous research has addressed the potential for ECM suppression through solder pad design modifications and a comparison between solder mask-defined and copper-defined pads. Our previous study [39] showed that the solder pad design could affect flux spattering during the reflow process. This prompted the question of whether the different designs of the pads could also affect the ECM.

## 2. Materials and Methods

### 2.1. Materials

The experiments were performed on single-sided FR4 boards with copper conductive pads. The copper pads with a thickness of 18 μm were covered with a hot air solder leveling (HASL) surface finish composed of SN100C^®^ solder alloy from Balver Zinn (Balve, Germany) (SnCu0.7Ni0.06Ge). The tested PCBs had dimensions of 25.5 × 80.5 mm and a thickness of 1.5 mm. The PCB comprised seven pairs of comb pattern electrodes, the design of which was in accordance with the IPC-TM-650 2.6.3.3 standard [40]. This standard defines the thickness of the lines as 0.4 mm and the spacing between them as 0.5 mm.

**Figure 1:**
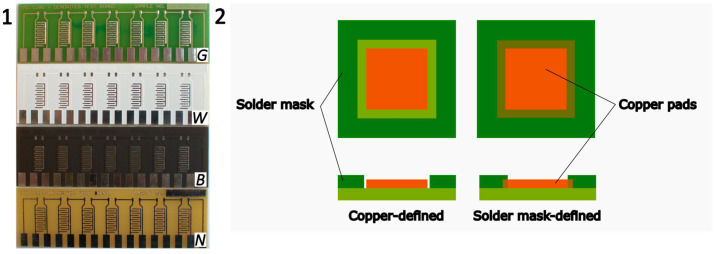
Tested PCBs: 1—different solder masks (G—green, W—white, B—black) and without solder mask (N—none), 2—different types of copper pad designs.

The PCBs were designed with and without a solder mask, and three types of solder masks were employed. The three used types of solder masks (see Figure 1) can be distinguished by their color: green (G), white (W), or black (B). The application of the three photoimageable solder masks was performed by screen printing. For all the solder masks, demineralized water and Na_2_CO_3_ were used for the cleaning and developing processes, respectively. The primary differences in their application lay in the ratio of components to a hardener and in the pre-drying temperatures. Apart from these variations, the application process for each solder mask remained identical and should not affect their surface morphology.

For each solder mask, two different designs of the copper pads were tested. The first design is called copper-defined, where the copper pads are defined by their own dimensions, and there is a narrow gap between the solder mask and copper. The second design is the solder mask-defined. In this case, the copper pad is wider than in the previous case; the solder mask is in direct contact with the copper pads covering them on the sides. In this paper, the copper-defined design will be referred to as CD, and the solder mask-defined as SD. The width of the CD pads is 0.4 mm and the insulation gap between each pad is 0.5 mm. In the case of SD, the width of the copper pad is greater, yet the solder mask extends to cover its sides, so the visible and uncovered copper area is the same as in the previous case. The width of the insulation gap (solder mask between the electrodes) is also 0.5 mm.

**Figure 2:**
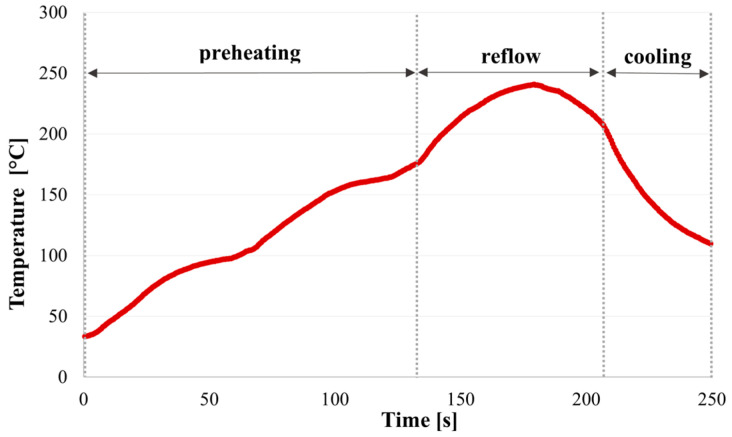
Reflow profile.

In half of the test PCBs, lead-free, no-clean solder paste was applied and reflowed using the conventional soldering process. The solder paste utilized in this study contained Sn95.75Ag3.5Cu0.75 solder alloy and no-clean flux designated as ROL0 in accordance with the J-STD-004 standard [41]. The solder paste labeled M31-GRN360-K1MK-V4 was supplied by Senju Manufacturing Europe s.r.o. (Prague, Czech Republic) and was deposited by screen printing and underwent a reflow process in the forced air convection reflow oven, Mistral 260 (Spidé, Harderwijk, The Netherlands). After the reflow process, the no-clean flux residues were left on the PCBs without cleaning. The used reflow temperature profile for soldering can be seen in Figure 2.

All the tested combinations are summarized in Table 1. The samples are referenced by a combination of letters. For example, the sample with a copper-defined design, green solder mask, and HASL surface finish is referred to as CD_H_G.

### 2.2. Methods

**Figure 3:**
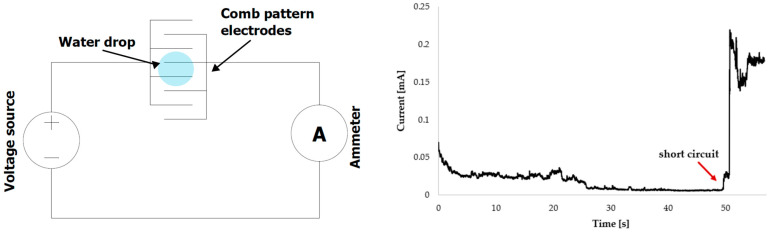
WDT schematic—**left**, current time dependence (TTF determination)—**right**.

The water drop test (WDT) and thermal humidity bias test (THBT) were performed to evaluate electrochemical migration. Although the THBT method is designed to reflect real-life conditions, the WDT was also included in the measurements. As surface roughness has been demonstrated to influence condensation [25,36,37], the WDT test should be valuable in more accurately identifying the impact of the tested factors on other ECM phases, such as anodic dissolution, ion transport, and dendrite growth. The setup of the water drop test can be seen in Figure 3. The 5 µL drop of high-purity water was placed on the PCB with a precise pipette, with the objective of ensuring that the drop was consistently placed in the same location in order to achieve a comparable coverage area. The conductivity of the water utilized was quantified at 6.5 µS/cm. The experiment was performed at an ambient temperature of 23 ± 1 °C while the bias voltage of 10 VDC was applied, and the current between the electrodes was measured. The current-time record was used to determine the time to failure (TTF), the time when a short circuit occurs and is recognized as the first current surge (see Figure 3). As the test devices for WDT, voltage source 3631A (HP Inc., Palo Alto, CA, USA) and multimeter 34410A (Agilent, Santa Clara, CA, USA) were employed. Both test devices were connected to the computer and controlled by the LabView program. The dendrite growth was also monitored by an optical microscope (SMART) during WDT.

A thermal humidity bias test was performed in a climate chamber CT-40/200 (CTS CmbH, Hechingen, Germany). The experimental parameters were adapted from the international standard IPC-TM-650 (2.6.14D) [42], which specifies a testing temperature of 85 °C, a relative humidity of 90%, and a test duration of 168 h. The standard recommends an applied bias voltage of 10 VDC. However, preliminary testing revealed that no dendrite structures were observed after the suggested time duration. Consequently, it was decided to increase the voltage for all tests to 20 VDC in order to promote the ECM process. During the test, the current between electrodes was also recorded to determine TTF, and the same measurement devices as for WDT were used.

Dendrite structures were subsequently observed with a scanning electron microscope (SEM) Phenom ProX (ThermoFisher Scientific, Waltham, MA, USA). Energy-dispersive X-ray spectroscopy (EDX) was employed to identify the elements comprising a given dendrite, thereby determining the migrating metal, and was conducted concurrently with the SEM examination.

In order to better differentiate between the three types of solder masks and understand their characteristics, several measurements were performed. Their surface roughness was measured on a confocal microscope VK-X1000 (Keyence, Osaka, Japan) in the profile/line (Ra, Rz, Rq) and on a surface area (Sa, Sz). Ra (Sa) is the arithmetic mean of the absolute values of the surface height deviations from the mean value in a given measured profile (area). Rz (Sz) determines the span from the maximum height to the maximum depth of the profile along the entire measured profile (area). The Rq, also known as RMS, is the root mean square of the height deviations from the mean line over the measured length. Texture aspect ratio Str, which expresses the degree of isotropy of the surface texture, was also determined.

A wettability measurement was conducted to evaluate the hydrophobic/hydrophilic properties of the surfaces under investigation. A 5 µL droplet of distilled water was placed on the test PCBs, captured with the microscope from the side view, and the contact angle was subsequently measured. This process was repeated twelve times for each solder mask and bare FR4.

Since the manufacturer did not disclose the chemical composition of the solder masks, we conducted Fourier-transform infrared spectroscopy (FTIR) and EDX analysis. FTIR analysis was performed using a VERTEX 80 spectrometer (Bruker Corporation, Billerica, MA, USA) to mainly determine the organic components of the different solder masks. EDX analysis was employed to identify the additives within the solder masks. For a comprehensive comparison, both analyses included a bare FR4 board.

## 3. Results

### 3.1. Surface Parameters

**Figure 4:**
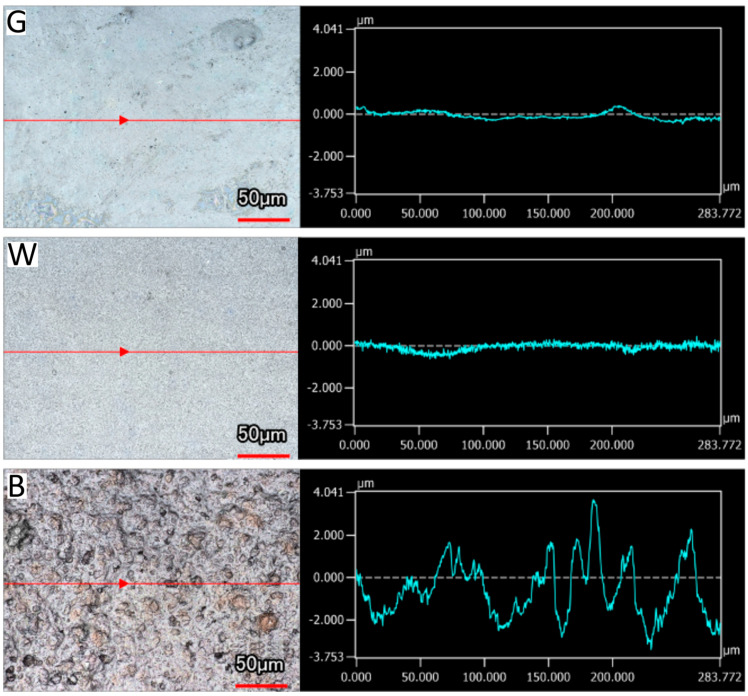
Surface roughness of green (G), white (W), and black (B) solder masks.

The results of surface roughness measurement are presented in Table 2, and an example of surface roughness analysis for each type of solder mask is displayed in Figure 4. The black solder mask exhibited the greatest surface roughness. The surface roughness of the green and white solder masks was found to be highly comparable.

**Figure 5:**
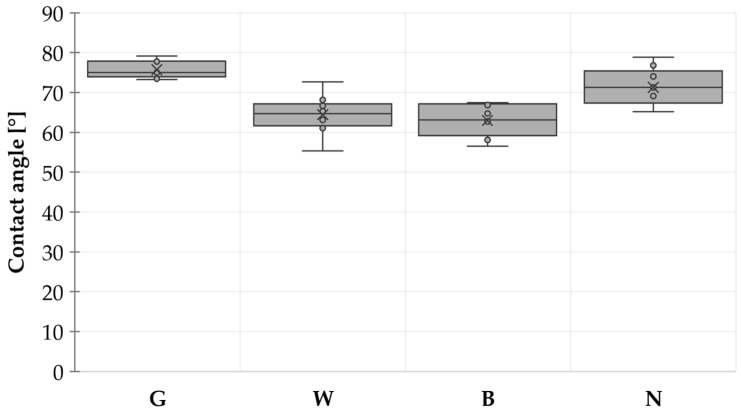
Contact angle.

The results of the wettability test demonstrated that, in all cases, the contact angle for the solder mask was less than 80°, indicating the hydrophilic nature of the surfaces (Figure 5). Both the white and black solder masks exhibited similar wettability, while the green solder mask demonstrated a slightly higher contact angle comparable to the surface of FR4 board. Despite this difference, all tested solder masks and the bare PCB are considered hydrophilic.

### 3.2. Solder Mask Composition

**Figure 6:**
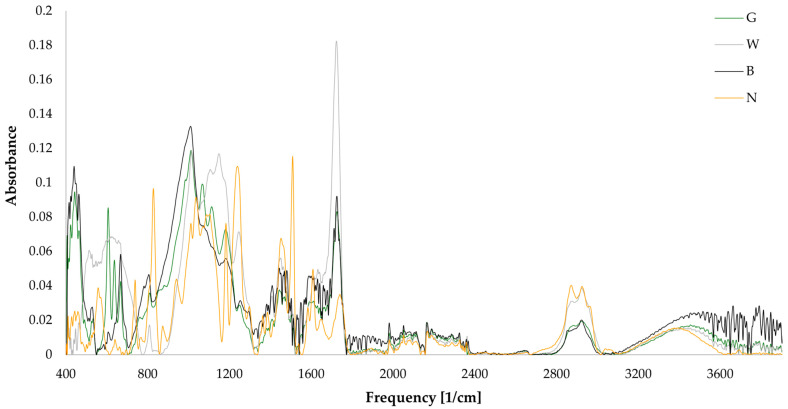
FTIR spectrum.

The spectra obtained from the FTIR analysis are presented in Figure 6. The data were interpreted with the assistance of the evaluation software Bruker OPUS 8.8.4 and available literature [43,44,45,46]. It indicates that the FR4 is composed mainly of epoxy resin. The spectral range from 3600 cm⁻^1^ to 1800 cm⁻^1^ exhibited consistent characteristics across all samples, with slight fluctuations in peak intensities around 2900 cm⁻^1^. In particular, the wide peak observed at 3500 cm⁻^1^ is attributed to the hydroxyl group (O–H) in the epoxy resin or urethanes, while the peaks at 2900 cm⁻^1^ are associated with the methyl (C–H) group. However, due to the presence of noise from CO_2_ and the diamond crystal employed in the measurement, the range from 2600 cm⁻^1^ to 1800 cm⁻^1^ was excluded from the evaluation. A prominent feature was a sharp peak at 1720 cm⁻^1^ across all solder masks, indicating the presence of a C=O compound. The white solder mask exhibited a peak that was twice as high as those observed in the black and green solder masks. C=C stretching is observed at 1640 cm^−1^ and 1450 cm^−1^. The peak at 1020 cm^−1^ is indicative of a C–O stretch, and C–H bending appears at 810 cm^−1^. The spectral region around 690–515 cm⁻^1^, which is prominent in the white solder mask, may indicate the presence of C–Br bonds. The green and black solder masks display more distinct peaks around 670 cm⁻^1^, which may be indicative of sulfate content. Furthermore, the peak observed at approximately 460 cm⁻^1^ is indicative of the presence of Si–O bonds, most likely from the silicon dioxide filler. The observed variations in solder mask composition are primarily attributed to the different additives and fillers, as evidenced in the fingerprint region (1500–400 cm⁻^1^). While the non-coated FR4 matches with epoxy resin in the polymer database, the green, white, and black solder masks match polyurethanes, which is confirmed by the presence of characteristic FTIR signals as well as nitrogen content detected by EDX.

**Figure 7:**
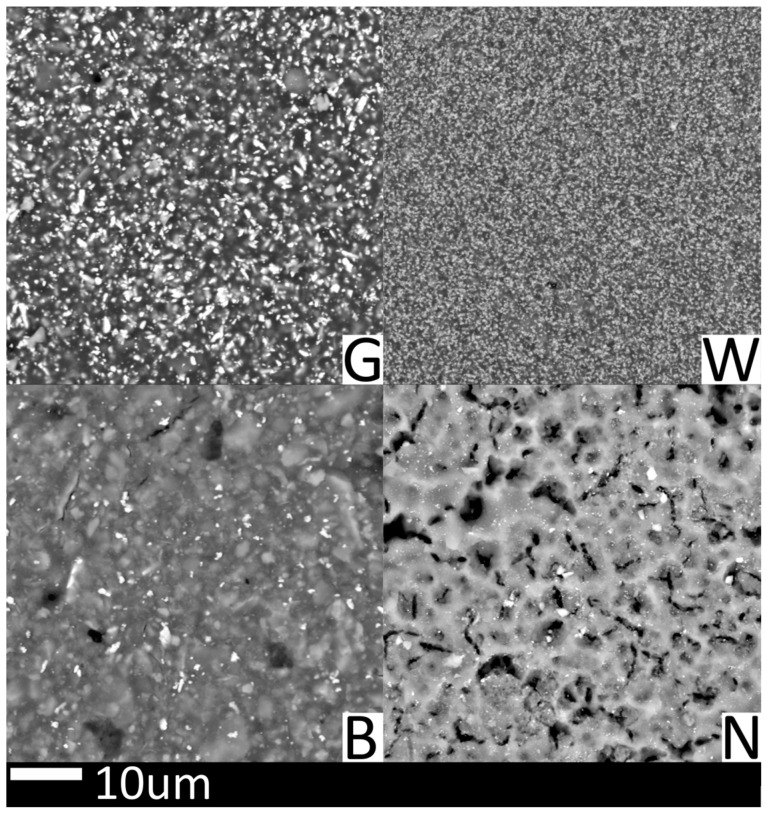
Close-ups of solder masks and FR4 on SEM.

Given the differences in composition between the solder masks, primarily in their fillers, SEM analysis was conducted to obtain more precise results (Table 3). All test variants were found to contain carbon, oxide, and silicon. The green and black solder masks exhibited a strikingly similar composition, with the exception of the presence of bromine in the black solder mask, which serves as a flame retardant. The silicon content is slightly higher in the black solder mask, which may be the cause of the higher surface roughness. In contrast, the white solder mask contained titanium, which is responsible for its white pigmentation, and calcium, which enhances mechanical strength. Elements such as magnesium, bromine, silicon, barium, and sulfur can originate from compounds such as BaSO_4_, BaTiO_3_, SiO_2_, and Mg_3_Si_4_O_10_(OH)_2_, which improve thermal resistance and mechanical properties [47,48,49,50]. The elements identified on the bare FR4 were limited to carbon, oxide, silicon, bromine, and chloride. As illustrated in Figure 4 and Figure 7, the discrepancy in surface roughness observed among solder masks can be attributed to the size of filler particles present within each mask. The black solder mask contains the largest particles, while the white solder mask comprises particles of a markedly smaller size.

### 3.3. Results from the Water Drop Test

**Figure 8:**
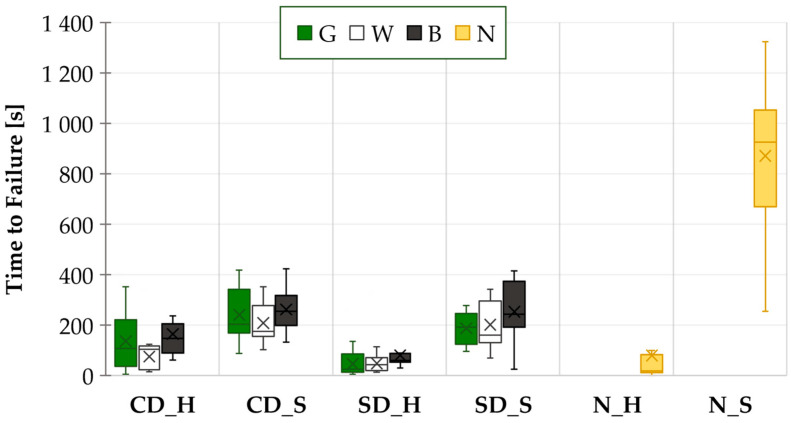
WDT results—TTFs for all the tested combinations.

A water drop test was conducted ten times for each variant. The results for all the tested combinations, including the results for the combination without a solder mask, are presented in Figure 8 in the form of a boxplot.

HASL-coated samples with the black solder mask consistently exhibited the longest mean time to failure (MTTF) across all cases (CD_H, SD_H). The time to failure was observed to be approximately 75% longer on average when the rough (black) solder mask was used instead of the smooth (white) one. Regarding the green and white solder masks, the difference in their TTFs was not as pronounced in comparison to the black solder mask.

The influence of the copper pad design is most evident in the HASL samples. The copper-defined design (CD_H) demonstrated longer TTFs than the solder mask-defined design (SD_H) across all mask types tested. Additionally, the TTFs for CD_H displayed a greater degree of dispersion in the obtained data compared to those for SD_H.

The results for samples with SAC solder alloy were found to be consistent with those of the HASL results. It was confirmed that the longest mean time to failure (MTTF) values were observed with the black solder mask. The lowest MTTF was observed for samples with a copper-defined design and a white solder mask (CD_S_W) and for samples with a solder mask-defined design and a green solder mask (SD_S_G).

The difference between the CD_S and SD_S was less pronounced than that observed between the HASL surface electrodes. The higher ECM resistance of CD_S was not as evident as it was with HASL surface electrodes. The sole discernible distinction was observed in the case of the green solder mask. The copper pad design exhibited minimal influence on ECM when the black and white solder masks were tested.

Comparing electrode types, the MTTFs were consistently shorter for samples with the HASL electrode than with the SAC electrode. The samples with no solder mask and applied solder paste exhibited the greatest TTF values. In this case, the absence of a solder mask and the presence of flux residues led to a synergistic effect and ECM suppression. On the other hand, the samples without the solder mask and HASL surface finish (N_H) demonstrated a markedly reduced ECM resistance compared to the N_S samples and were comparable to SD_H samples.

**Figure 9:**
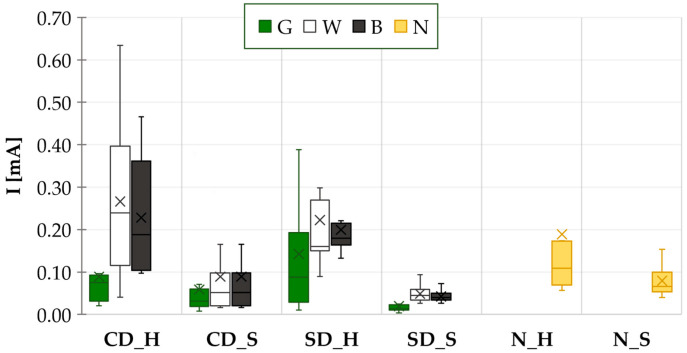
WDT results—base leakage current.

**Figure 10:**
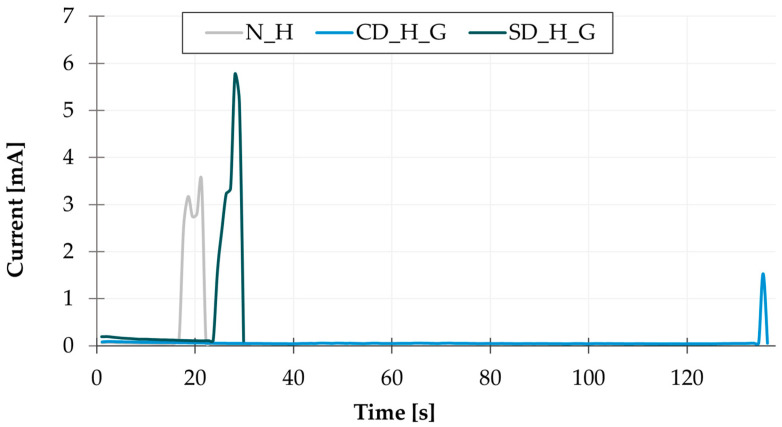
An example of current measurements—WDT.

A comparison of the base leak current values (Figure 9) measured during WDT revealed that the green solder mask exhibits the lowest values, while white and black solder masks exhibit values that are comparable and significantly higher in comparison to the green solder mask. The correlation between the base leakage current and TTF is minimal. However, it can be observed that the base leakage current measured on the HASL electrodes is higher than on the SAC electrodes, where the flux residues act as an insulating layer. This is accompanied by a longer TTF measured on the SAC samples. An example of the current measurement during WDT is displayed in Figure 10.

### 3.4. Results from the Thermal Humidity Bias Test

**Figure 11:**
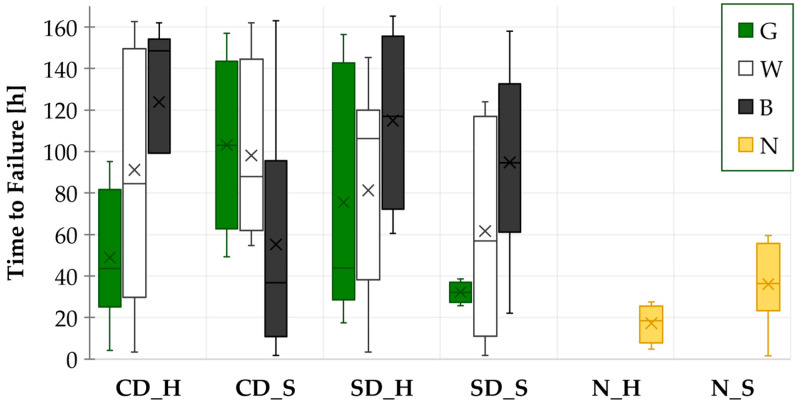
THBT results—TTFs for all combinations.

The results from all the THBT measurements can be seen in Figure 11. The influence of the copper pad design is not particularly evident from the results; however, in four out of six test combinations, the longer TTFs were measured on samples with the CD design.

The test results exhibited a high correlation with those obtained by the WDT. Except for the CD_S samples, the longest TTFs were observed with the black mask. In the case of CD_H, SD_H, and SD_S test samples, the shortest TTFs were observed with the green solder mask, followed by the white solder mask.

A comparison of HASL and SAC samples revealed that, in contrast to the results obtained from WDT, the TTF values from THBT showed no discernible differences.

**Figure 12:**
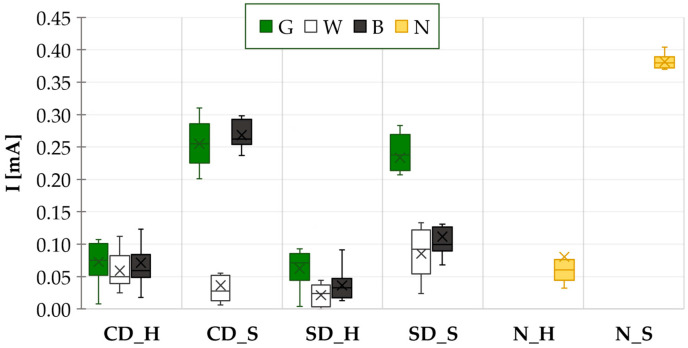
THBT results—base leakage current.

The correlation between base leakage current from the THBT (Figure 12) and TTF is also minimal. The data from the THBT shows that the lowest current values were observed in each case with the white solder mask, while the highest values were noted mostly with the green solder mask. There appears to be a discrepancy between the current values from THBT and the WDT, which suggests the opposite.

**Figure 13:**
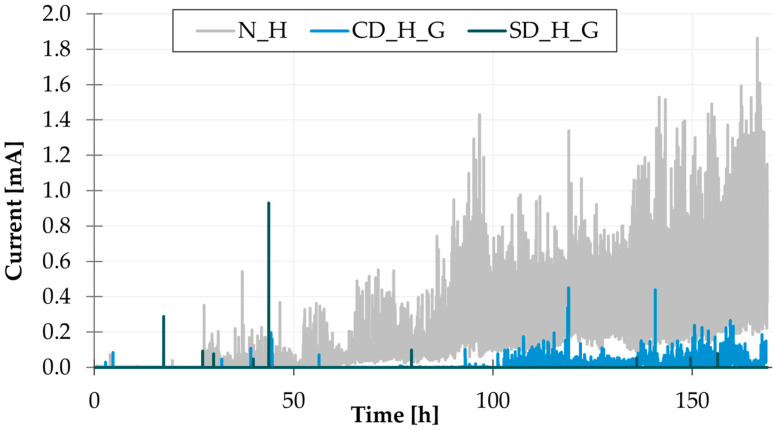
An example of current measurements—THBT.

The THBT results indicate that the solder mask inhibited the ECM in comparison to a scenario where the solder mask was not applied. An example of the current measurements of PCBs with green solder mask and without solder mask (both with copper pad design) is displayed in Figure 13. As evidenced by the graph, the samples without a solder mask exhibit reduced ECM resistance in comparison to samples with a solder mask. The influence of the presence of the solder mask on ECM suppression was significant for all solder masks tested. The intensity and frequency of short circuits on the PCBs were significantly higher when the solder mask was not applied. Although the presence of a solder mask did not entirely prevent the short circuit, the graph clearly indicates that the presence of a solder mask is beneficial for ECM suppression and reducing the short circuit current.

### 3.5. SEM and EDX Analysis

After each test, the tested PCBs were examined by the SEM, and EDX analysis was performed on dendrites. The EDX results are shown in Table 4, where migrating metals are identified for each test combination. The presence of tin was found in all of the cases. During the water drop test, tin was the only migrating metal regardless of the electrode type except for two variants, CD_H_W and SD_H_B, where copper was also found. After THBT, the presence of copper in dendrites was observed to be more frequent. The migration of tin and copper was found in most cases. Silver, as one of the migrating elements, was only found in dendrites on sample N_S.

**Figure 14:**
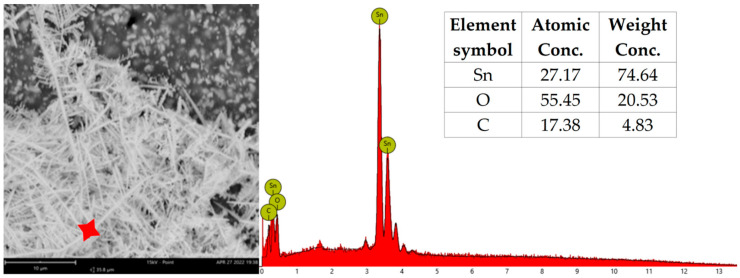
An example of EDX analysis results—CD_H_G–WDT.

**Figure 15:**
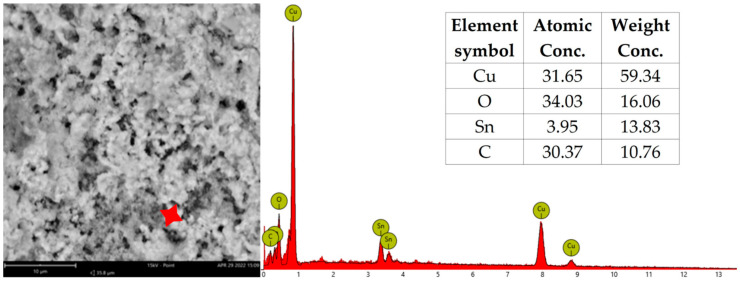
An example of EDX analysis results—CD_H_G–THBT.

In Figure 14 and Figure 15, the example spectrum from the EDX analysis is depicted. The presence of oxygen suggests the formation of metal oxides, whereas carbon probably originated from the substrate and formed precipitates from the electrolyte or impurities. The analyzed dendrites after THBT did not exhibit the typical “tree” structure due to the extended duration of the test compared to WDT.

## 4. Discussion

### 4.1. The Influence of the Solder Mask

The differences in the solder mask composition have been found mainly in their fillers and additives. Faulty manufacturing processes, such as inadequate solder mask curing [38], can result in the presence of unstable compounds, which could increase contamination and the risk of electrochemical migration. Unless there was a problem with the production of the PCBs, which is not believed to be the case, there should not be any additional contamination affecting ECM.

Differences in the composition of the solder masks and the sizes of particles may have influenced their surface roughness, which influences the wettability of the solder mask. However, the wettability of the black and white solder masks was found to be more similar to that of the green solder mask. This suggests the presence of another factor that significantly influences wettability, specifically the composition of the solder mask. As different materials may have different surface energies, different compositions of the solder mask would influence the wettability of the surface as well. Therefore, the wettability is affected by the combination of these factors. The influence of different compositions on surface wettability has been eliminated during the WDT.

**Figure 16:**
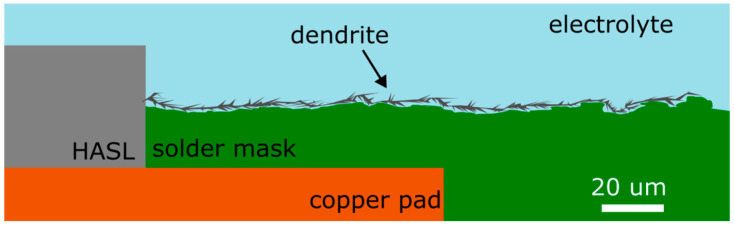
Dendrite growing over rough solder mask (solder mask-defined).

The results of the THBT experiments with HASL-coated electrodes suggest that the use of a black solder mask with a higher surface roughness is more suitable for suppressing electrochemical migration, thus prolonging the mean time to failure more than other tested solder masks. The results of the SD_S test also lend support to this conclusion. CD_S, however, exhibited a contrasting behavior, which is likely attributable to the influence of more potent factors, such as the impact of flux residue. In contrast with our finding that increased surface roughness hinders the ECM, previous studies indicate that higher surface roughness should enhance moisture condensation due to improved wettability [25,36]. However, the experimental results obtained in this study suggest that the surface roughness between the electrodes may also affect ion transport and dendrite growth through the formation of a mechanical barrier (see the illustration in Figure 16). The effect of the mechanical barrier caused by the solder mask surface roughness may have prevailed over the increase in the rate of condensation. The conclusion has been supported by the WDT. While some variants exhibit less noticeable differences, particularly the copped defined pad, overall, the mean values of TTFs were the longest with the black solder mask. Consequently, it seems that the black solder mask is the least susceptible to ECM out of all the tested solder masks.

The susceptibility to ECM was observed to be higher for samples without any solder mask, which is in agreement with previous studies [10,25]. The higher surface roughness of the FR4 might have enhanced the formation of a uniform, homogeneous electrolytic layer, allowing the movement of ions, compared to the smoother surface of the solder masks. The condensation rate here prevailed over the effect of the mechanical barrier caused by the surface roughness. Furthermore, the solder mask provides an additional mechanical barrier against the formation of dendrites.

The only exception was observed during the WDT test for the sample without a solder mask but with applied solder paste on the electrodes. This sample showed much longer TTF than any other sample. The root cause was most likely due to flux residues between the electrodes, which adhered more effectively to the PCB surface without a solder mask and created a barrier. The barrier on the PCB without the solder mask took longer to break through than in the samples with the solder masks. In the case of THBT, higher ambient temperatures may have contributed to faster barrier disruption, while its effect may have also been reduced over the longer test period.

### 4.2. The Influence of the Design of the Copper Pads

The WDT results suggest that the copper-defined solder pad design is more suitable for suppressing ECM. Considering the results of this work, where dendrite growth was hindered by higher solder mask surface roughness (with a maximum range of around 5 µm in our case), incorporating a gap next to the copper pad followed by a solder mask at least 20 µm high should also impede its growth.

After the application of the solder paste, the distinctions between the design variants became less apparent. The influence of the copper pad design may be undermined by flux residues, which have the potential to create a barrier inhibiting the anodic dissolution, ion transport, and dendrite growth. Additionally, flux residues may occupy the gap between the pad and the solder mask.

As for the THBT, other factors, such as high test temperature or condensation, may have had a more pronounced effect on the results than the effect of the copper pad design. The condensation phase, in particular, could have had a significant effect on the ECM, more than the effect of the design, which became crucial mostly during the dendrite growth itself.

### 4.3. The Influence of the Electrode Type

The WDT test demonstrated a clear correlation between electrode type and ECM. HASL-coated electrodes exhibited a lower ECM resistance compared to the electrodes with SAC solder paste. The primary reason for the longer TTFs with SAC samples was most likely the presence of flux residues, which have been demonstrated to suppress the ECM [23]. Flux residues create a mechanical barrier that impedes access of the electrolyte to the electrodes, thereby inhibiting electrode dissolution and decelerating ion transport and dendrite growth, which have to penetrate to reach the opposing electrode. Furthermore, the different elemental compositions of the HASL surface finish and SAC solder paste can influence the TTF. The presence of silver or different concentrations of elements within the electrode materials could hinder ECM. Previous studies [33,51,52] have shown that varying ratios of copper and silver within tin-based solder alloys affect ECM differently.

The results of the THBT did not demonstrate a discernible correlation between TTF and electrode material. Therefore, the WDT observation, where the longer TTF was observed on HASL-coated electrodes, does not apply here. The test conditions may have diminished its influence as well as the condensation process or the influence of flux residues, as higher temperatures during the test could cause the flux residues to change form and soften the barrier.

The primary migrating element was tin in all cases. Nevertheless, the presence of copper was also identified on samples that underwent THBT. The copper is likely to have originated from the base copper pad, as it was found in both HASL and SAC samples. The amount of copper released from SAC alloy would be very small, as stated by Yu et al. [33].

The differences in dendrite composition between WDT and THBT are primarily attributable to the duration of the test but also to the higher voltage in THBT. In THBT, increased electrode corrosion occurred, involving not only the migration of tin but also other metals present in the alloy or the electrode material itself. During the longer test, the copper electrode could have been exposed, leading to its dissolution and migration. THBT is designed to operate for a certain duration, during which it is susceptible to an increased number of short circuits and significant migration. In contrast, WDT is a quick diagnostic tool that is terminated after the initial short circuit.

The silver present in the case of sample N_S must be derived from the SAC alloy, particularly from the SnAg intermetallics present in the solder volume. This is in accordance with the findings of Zhong et al. [53]. In this case, the migration of silver can be attributed to the synergistic effect of the absence of the solder mask and longer exposure during THBT to a humid environment and higher voltage. Consequently, even silver present in the intermetallic compounds had enough time to dissolve.

### 4.4. Comparison of WDT and THBT Results

Given the disparate nature of the conducted ECM tests (WDT, THBT), it was to be anticipated that their results would diverge.

The WDT results indicated an apparent difference between the HASL and SAC-coated electrodes, with HASL electrodes demonstrating a higher susceptibility to ECM in comparison to SAC electrodes. Furthermore, the WDT suggested that solder mask-defined designs with HASL electrodes exhibited greater susceptibility to ECM than copper-defined designs.

In contrast, both trends were not observed in the THBT results. The WDT does not include a condensation step, thereby eliminating the impact of surface roughness on the initial stage of ECM and instead focusing on the subsequent stages. In contrast, the THBT method more closely approximates real-world conditions, including the formation of an electrolyte layer and the influence of surface roughness and the solder mask composition on this process. It can be stated that the condensation and higher test temperatures in THBT had a more pronounced impact on ECM than the factors being tested, such as solder pad design and the type of electrodes. This was particularly evident with SAC-coated electrodes, where flux residues play an essential role. The mechanical barrier formed by flux residues exhibited different behavior during THBT compared to that observed in WDT.

The higher test temperature and voltage in THBT may have facilitated the disruption of the barrier to a greater extent, and the discrepancies in the results attributable to the barrier may have diminished over the extended test duration. For instance, the pronounced disparities between the N_H and N_S observed in WDT outcomes were less prominent during THBT.

## 5. Conclusions

A comprehensive investigation of the influence of solder mask types, pad design, and electrode material in terms of ECM has been carried out using WDT and THBT. The following conclusions have been reached based on the presented evidence.

- Solder mask-defined pad design appears to be more prone to ECM, as the copper pad design inhibits the ECM in the majority of cases, due to the presence of the gap between the copper pad and solder mask.
- The results proved the importance of the use of the solder mask in preventing ECM.
- The longest MTTFs were mainly observed on the black solder mask, which had the highest surface roughness out of the tested variants, indicating that surface roughness exerts a substantial impact not only on the condensation phase, as stated in current literature, but also on the subsequent stages of ECM through the creation of a mechanical barrier.

It is advisable to consider the results of this work when designing PCBs, where feasible. In order to prevent the ECM, it is preferable to select a copper-defined pad over a solder mask-defined pad and to apply a solder mask with a higher surface roughness. These suggestions may result in a higher degree of reliability with respect to the ECM.

## Figures and Tables

**Table 1:** Tested combinations and their labels.

Copper Pad Design	Surface Electrode Material	Solder Mask Type
Copper-defined (CD)	HASL (H)	None (N)
Solder mask-defined (SD)	HASL + SAC (S)	Green (G)
		White (W)
		Black (B)

**Table 2:** Roughness parameters of the used masks and FR4 substrate.

Solder Mask	Ra [µm]	Rz [µm]	Rq [µm]	Sa [µm]	Sz [µm]	Str [-]
Green	0.16 ± 0.02	1.07 ± 0.16	0.20 ± 0.04	0.17 ± 0.02	7.40 ± 1.35	0.72 ± 0.09
White	0.15 ± 0.02	1.20 ± 0.07	0.18 ± 0.03	0.17 ± 0.03	4.98 ± 2.56	0.52 ± 0.18
Black	0.93 ± 0.08	5.20 ± 0.57	1.05 ± 0.15	0.99 ± 0.08	11.68 ± 3.35	0.61 ± 0.09
None	0.91 ± 0.12	5.41 ± 1.30	1.12 ± 0.15	0.89 ± 0.20	12.08 ± 3.57	0.85 ± 0.04

**Table 3:** Elements and their concentrations contained in the solder masks and bare PCB.

Solder Mask	Green		White		Black		None	
Element	Atomic	Weight	Atomic	Weight	Atomic	Weight	Atomic	Weight
C	30.89	17.24	32.35	23.21	25.99	13.99	76.18	55.97
O	35.78	26.60	38.42	36.72	37.38	26.80	17.87	17.49
N	20.84	13.57	19.81	16.58	20.68	12.98		
Ba	5.12	32.70	-	-	4.74	29.20	-	-
Si	2.40	3.13	1.36	2.29	6.00	7.55	0.52	0.89
S	3.15	4.70	0.24	0.46	2.86	4.11	-	-
Mg	1.82	2.05	0.33	0.48	1.21	1.32	-	-
Ti	-	-	6.44	18.43	-	-	-	-
Br	-	-	0.86	1.39	1.13	4.05	5.09	24.90
Ca	-	-	0.18	0.44	-	-	-	-
Cl	-	-	-	-	-	-	0.34	0.74

**Table 4:** EDX analysis results—dendrite composition found metals on samples after WDT and THBT.

Solder Mask Type	Pad Design	Electrode Type	WDT	THBT
Green	CD	H	Sn	Sn, Cu
		S	Sn	Sn, Cu
	SD	H	Sn	Sn, Cu
		S	Sn	Sn, Cu
White	CD	H	Sn, Cu	Sn, Cu
		S	Sn	Sn, Cu
	SD	H	Sn	Sn
		S	Sn	Sn
Black	CD	H	Sn	Sn, Cu
		S	Sn	Sn
	SD	H	Sn, Cu	Sn, Cu
		S	Sn	Sn, Cu
None		H	Sn	Sn, Cu
		S	Sn	Sn, Cu, Ag

## Data Availability

Data is contained within the article.
